# Industrial Validation and Mechanical Characterization of SMA Mixtures Stabilized with Recycled Polymeric Fibers from Waste Tires

**DOI:** 10.3390/polym18020156

**Published:** 2026-01-07

**Authors:** Alejandra Calabi-Floody, Gonzalo Valdés-Vidal, Cristian Mignolet-Garrido, Cristian Díaz-Montecinos, Claudio Fonseca-Ibarra

**Affiliations:** 1 Department of Civil Engineering, Universidad de La Frontera, Temuco 4780000, Chile; 2 GiPAV–Grupo de Investigación en Pavimentación Vial, Temuco 4811230, Chile; 3 CDI–Centro de Desarrollo e Investigación, Santiago 7810000, Chile; 4 ISA Vías, Santiago 8320000, Chile

**Keywords:** stone mastic asphalt (SMA), waste tire, waste tire textile fibers, polymeric additive, performance properties, industrial production, ELT

## Abstract

This study investigates the industrial validation of a granular additive derived from waste tire textile fibers (WTTF) developed to replace the conventional cellulose stabilizing additive in stone mastic asphalt (SMA) mixtures while enhancing their mechanical performance. Building on previous laboratory-scale findings, this work evaluates the feasibility and mechanical behavior of this recycled-fiber additive under real asphalt-plant production conditions, advancing a sustainable solution aligned with circular economy principles. Three asphalt mixtures were fabricated in a batch plant: a reference SMA (SMA-R) containing a commercial cellulose additive, an SMA incorporating the WTTF additive (SMA-F), and a reference hot mix asphalt (HMA-R). The WTTF additive was incorporated in a 1:1 proportion relative to the cellulose additive. Performance was assessed through tests of cracking resistance (Fénix test), stiffness modulus, fatigue resistance (four-point bending test), moisture susceptibility (ITSR), and resistance to permanent deformation (Hamburg wheel tracking). Industrial validation results showed that the SMA-F mixture met the design criteria and achieved superior mechanical performance relative to the reference mixtures. In particular, SMA-F exhibited greater ductility and toughness at low temperatures, reduced susceptibility to moisture-induced damage, and higher fatigue resistance, with an increase in fatigue durability of up to 44% compared to SMA-R. The results confirm that the WTTF additive is both feasible and scalable for industrial production, offering a solution that not only improves pavement mechanical performance but also promotes the valorization of a challenging waste material.

## 1. Introduction

Asphalt pavements are among the most commonly employed solutions in global road infrastructure [1]. Hot mix asphalt (HMA) mixtures are extensively employed in flexible pavement construction, distinguished by their ability to endure cyclic loading and perform well under a wide range of environmental conditions [1,2,3,4]. In Europe, the annual production of these mixtures exceeds 260 million tonnes [5], which reflects their widespread application and significance to the road sector. These mixtures are composed predominantly of mineral aggregates (≈95%) [4,6] and asphalt binder (≈5%), which acts as a binding and waterproofing agent between the aggregates, providing durability to the mixture [7,8]. The mechanical performance of asphalt mixtures is largely determined by the temperature dependence of the asphalt binder and the loading conditions to which the pavement is subjected [9,10]. At low temperatures, the asphalt binder behaves as a solid-brittle material, becoming susceptible to cracking when stresses exceed its deformation capacity [11,12,13]. At intermediate temperatures, the asphalt binder demonstrates viscoelastic behavior, which enables partial recovery from the applied stresses [12]. However, a portion of the deformations generated within this range progressively accumulates as permanent deformations [12,14,15]. Under repeated loading, the accumulation of these deformations promotes the onset of fatigue damage, compromising pavement durability [13]. At high temperatures, the asphalt binder loses stiffness and behaves predominantly as a viscous material, reducing its recovery capacity and promoting the development of permanent deformations [12,13,16]. Therefore, the thermal susceptibility of the asphalt binder compromises the durability of asphalt mixtures, particularly under demanding traffic and environmental conditions.

In this context, stone mastic asphalt (SMA) mixtures emerge as a high-performance technical alternative, providing enhanced structural and functional resistance [17,18,19,20]. They are characterized by a gap-graded particle size distribution with a robust mineral skeleton and elevated asphalt binder content, generally 6.0–7.0% [18,19,20,21]. Such properties provide SMA mixtures with enhanced mechanical performance and durability relative to HMA. To prevent binder drainage and maintain mixture stability, stabilizing additives are employed, primarily cellulose fibers, as well as mineral fibers and polymers [19,20,22,23,24].

Recently, attention has been focused on polymeric fibers derived from waste tire textile fibers (WTTF) as a potential alternative stabilizing material. These fibers are recovered during the recycling of end-of-life tires, typically through shredding, followed by the sequential separation of rubber, steel, and textile components [25]. The textile fraction generally constitutes between 1 and 10% of the total tire weight [26,27,28] and is predominantly composed of polyester and polyamides (nylon 6 and nylon 6.6) [28,29,30,31].

At present, the valorization of WTTF is limited, representing a significant challenge for the recycling industry [32,33,34]. Only a small fraction is currently used as fuel in cement plants, while the remainder is disposed of in landfills or incinerated [35,36]. This limited utilization is particularly concerning given that over 20 million tonnes of ELT are produced worldwide annually [37]. Based on these data, the annual production of WTTF is estimated to exceed 2.0 million tonnes, emphasizing the need to develop large-scale valorization strategies for this waste.

In this context, studies have evaluated the use of WTTF in asphalt materials, demonstrating significant effects on both the rheological behavior of asphalt binders and the mechanical properties of the mixtures. In particular, the inclusion of WTTF has been shown to enhance high-temperature performance, thereby increasing resistance to rutting and permanent deformation [38,39]. Additionally, different types of fibers have been investigated, showing improvements in the mechanical performance of asphalt mixtures [40,41,42,43,44]. According to Jia et al. (2023) [41], aramid, glass, and polyester fibers enhance the fatigue cracking resistance of asphalt mixtures. In contrast, polyester, polyamide, and carbon fibers improve the resistance of asphalt pavements to permanent deformation. Based on these studies, Valdés et al. (2022) [28] and Valdés et al. (2023) [45] developed a granular additive derived from WTTF for use in HMA and SMA mixtures. At the laboratory scale, the incorporation of this additive into HMA mixtures resulted in increased stiffness modulus, as well as enhanced resistance to permanent deformation and sensitivity to moisture [28]. In SMA mixtures, the design and performance properties of the SMA mixtures with WTTF additive were comparable to those of the reference SMA mixture with cellulose fibers [45]. Moreover, the additive enhanced fatigue resistance in both mixtures, increasing their capacity to withstand load cycles and extending their service life by over 100% [46]. From an environmental perspective, its use aligns with the principles of sustainability and circular economy, as it mitigates the impacts associated with asphalt mixture production while enabling the valorization of complex waste streams [47].

Although asphalt binder modification with polymers, rubber, and recycled waste tire textile fibers (WTTF) has been widely studied, limited research has focused on the industrial validation of these additives, particularly the polymeric fibers recovered from waste tires. This study addresses this gap by assessing the mechanical performance and production feasibility of stone mastic asphalt mixtures incorporating WTTF as a stabilizing additive under real manufacturing conditions. The findings provide evidence of the scalability of this sustainable additive, supporting its potential to enable more durable SMA mixtures and contributing to circular economy strategies in road construction.

## 2. Materials and Methods

**Figure 1:**
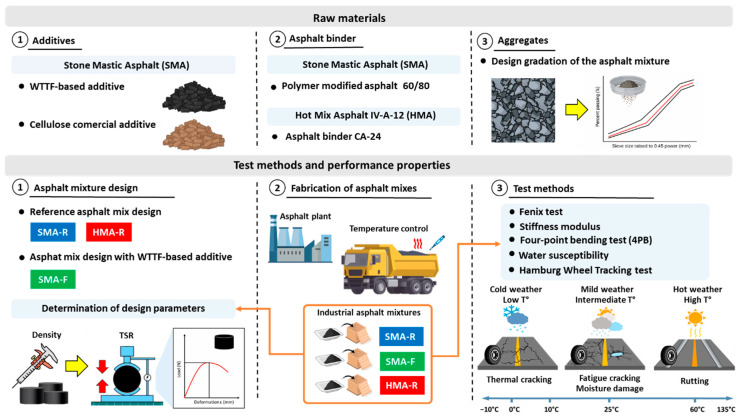
Flowchart of experimental design.

This chapter describes the materials, mixture designs, and experimental procedures used in this study to evaluate the performance of asphalt mixtures fabricated with a polymeric additive derived from waste tire textile fibers (WTTF). The experimental design implemented in this study is shown in Figure 1. In the first stage, two reference asphalt mixtures were designed and characterized in the laboratory: SMA-R and HMA-R, both following the criteria established by the Chilean standards for wearing courses. Additionally, a new SMA mixture (SMA-F) was developed, incorporating a polymeric additive (WTTF) as a replacement for the commercial cellulose additive used in the reference mixture. In the second stage, the WTTF-based polymeric additive was validated on an industrial scale. For this purpose, the selected mixtures were fabricated in a batch asphalt plant, maintaining the design parameters established in the laboratory. Representative samples were collected and stored for subsequent analysis. Finally, the properties evaluated were cracking resistance (Fénix test), stiffness modulus, fatigue resistance (four-point bending test, 4PB), moisture susceptibility, and resistance to permanent deformation (Hamburg wheel tracking test).

### 2.1. Materials

#### 2.1.1. Stabilizing Additives for SMA Mixtures

**Figure 2:**
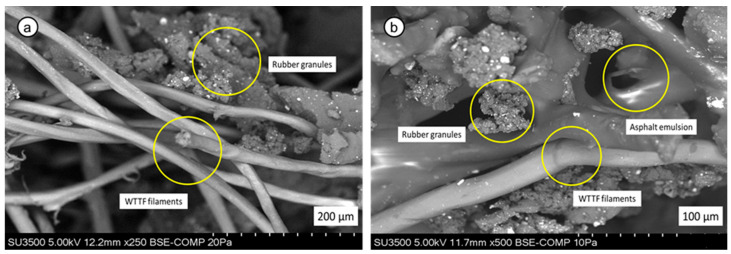
Morphological characterization: (**a**) SEM image of an as-received WTTF fiber; (**b**) SEM imagen of the WTTF-based additive.

SMA mixtures were fabricated using two stabilizing additives: (1) a commercial cellulose additive, commonly used in the industry; and (2) a novel polymeric fiber-based additive, patented in Chile [48] and Europe [49]. This innovative additive is composed of waste tire textile fibers (WTTF). As illustrated in Figure 2, the textile fibers exhibit rubber powder adhered to their surface. Their composition is predominantly polyester, as reported in previous studies [28,29].

**Figure 3:**
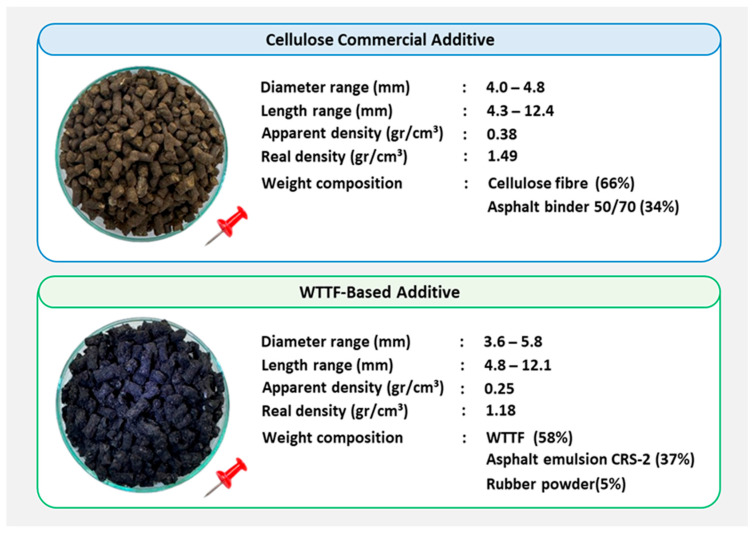
Characterization of the additives used in SMA mixtures [45].

The polymeric fibers were blended with a water-diluted asphalt emulsion in a 1:1:1 weight ratio. During the process, water evaporates as part of the emulsion breaking, allowing a uniform distribution of the asphalt emulsion over the polymeric fibers. The resulting raw material undergoes extrusion and cutting, producing the additive in granular form, which facilitates its integration into the SMA mixture. To prevent adhesion between additive granules during handling and application, rubber powder is incorporated at a 20:1 weight ratio. The physical characteristics of the additives used in this study are presented in Figure 3.

#### 2.1.2. Asphalt Binder

In this study, two types of asphalt binders were employed, classified according to the Chilean Standard [50]. For the SMA mixtures, a polymer-modified asphalt binder with a penetration grade of 60/80 Plus was used. Conversely, for the HMA mixture, a conventional CA-24 asphalt binder was employed. These binder selections reflect standard industrial practice in Chile, where pavement concessionaires specify the use of conventional binders for HMA and modified binders for SMA mixtures. The mixture designs are those requested by the concessionaires from the asphalt production plant where the production trials were conducted, ensuring that manufacturing conditions are representative of real practice. The properties of the asphalt binders used in the SMA and HMA mixtures are presented in Table 1 and Table 2, respectively.

#### 2.1.3. Aggregates

The aggregates used were of riverbed origin and contained, principally, particles of dolomite, basalt, dacite, andesite, rhyolite, sandstone, quartz, and quartzite, all conforming to the Chilean Standard for wearing courses [53]. Additionally, the SMA mixture included 9% mineral filler (lime) by weight of the aggregates. The properties of the aggregates used in this study are presented in Table 3.

**Figure 4:**
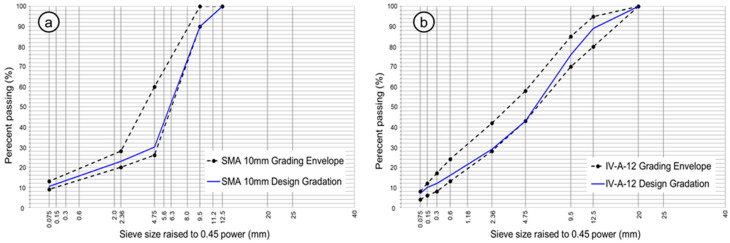
Design gradation: (**a**) SMA10 asphalt mixes; (**b**) IV-A-12 asphalt mixture.

The particle size distribution of the asphalt mixtures used in the experimental section was adjusted according to the Chilean standard. For the SMA mixtures, a grading band with a maximum nominal particle size of 10 mm (SMA10) was used [54], whereas for the HMA mixture, a semi-dense gradation corresponding to a type IV-A-12 mixture was adopted [53]. The aggregate gradations used in the SMA and HMA mixtures are shown in Table 4 and Figure 4.

### 2.2. Mix Design

The reference asphalt mixtures, SMA-R and HMA-R, were designed in accordance with the Chilean Standard [53]. The asphalt mixture designs used in this study were provided by the asphalt production company where the production trials were conducted. These mixture designs are those specified by pavement concessionaire companies for the construction and maintenance of their road networks in Chile and therefore reflect real industrial practice. The aggregate gradations, binder types, and stabilizing additives employed are those routinely used by the asphalt plant in its standard production processes. This ensures that the evaluated mixtures, both the reference SMA mixture and the one with WTTF-based polymeric additive as well as the reference hot mix asphalt (HMA), are representative of real construction conditions and meet the usual construction requirements.

The optimum asphalt content was determined as 7.3 ± 0.3 wt% of aggregates for SMA-R, and 5.1 ± 0.3 wt% for HMA-R. In this study, a novel polymeric fiber-based additive (WTTF) was incorporated as a substitute for the commercial cellulose fibers in SMA-R. The replacement was conducted on a 1:1 basis, equivalent to 0.5 wt% of the aggregate weight. The resulting mixture, referred to as SMA-F, was designed and characterized to match the volumetric properties and performance criteria of SMA-R.

Plant production of SMA-R, HMA-R, and SMA-F mixtures was conducted in accordance with laboratory-validated designs. The design parameters of each mixture were subsequently checked against the criteria defined by the Chilean standards to evaluate their capacity to preserve volumetric and mechanical properties under actual manufacturing conditions. Mixing temperatures were set at 176 °C for the SMA mixtures (SMA-R and SMA-F) and 152 °C for HMA-R, while compaction temperatures were 165 °C for SMA and 143 °C for HMA.

The design parameters evaluated included: stability, flow, air void content, voids in the mineral aggregate (VMA), voids in the coarse aggregate within the mixture $\left({VCA}_{MIX}\right)$, voids in the compacted coarse aggregate ${(VCA}_{DRC})$, and binder drainage.

### 2.3. Industrial Production of Asphalt Mixtures

**Figure 5:**
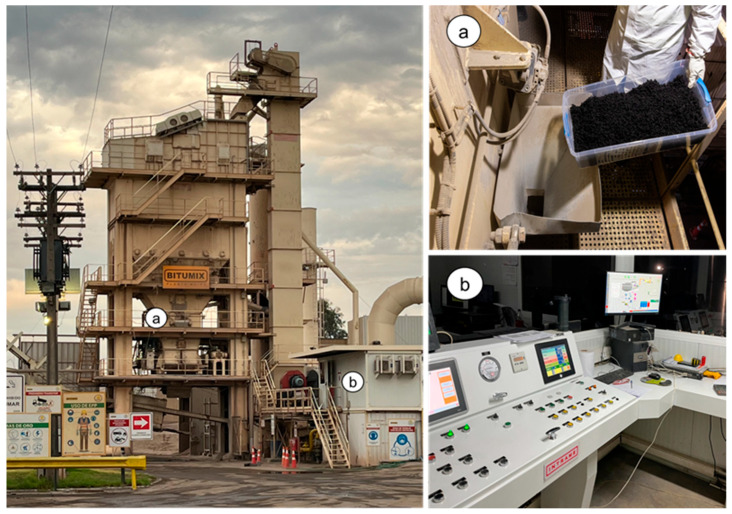
Batch plant used for the manufacture of asphalt mixtures: (**a**) area for the incorporation of the WTTF-based additive, where the additive is added to the mixture during plant production; (**b**) control tower where the addition of materials and the asphalt binder content are monitored.

**Figure 6:**
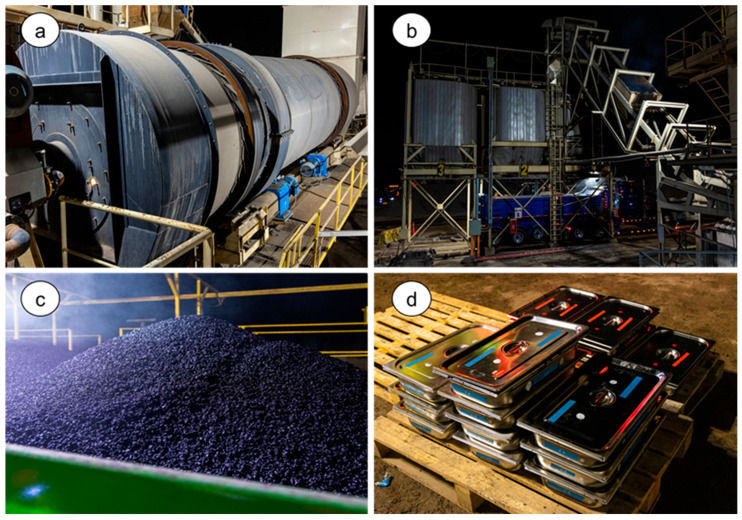
Overview of the industrial production process: (**a**) drum used for drying the aggregates before incorporation into the mixture; (**b**) transport of the produced mixtures to trucks for distribution; (**c**) control area where visual inspection and mixture temperature measurement are carried out; (**d**) storage of industrial mixtures for subsequent laboratory evaluation.

The asphalt mixtures were fabricated in a batch plant with a production capacity of 300 t/h. Aggregates were heated in a drying drum until reaching the mixing temperature specified in the design for each type of mixture. Once the target thermal conditions were achieved, the aggregates were separated by particle size using an automated dosing tower and fed into the mixing drum, where the preheated asphalt binder was incorporated at the established mixing temperature. The mixing process was maintained for 30 s to ensure proper homogenization and integration of the materials. The discharge temperature of the mixture was controlled using digital thermometers and infrared sensors. The fabricated asphalt mixtures were stored in metal containers to preserve their original conditions. In the laboratory, compliance with the design parameters was verified, and the mechanical behavior of the mixtures was evaluated through specific tests under controlled conditions, detailed in “Section 2.4. Testing methods”. Prior to evaluating each property, the samples underwent a single conditioning process to restore their workability. This involved heating the material to 110 °C for 3 h, followed by storage of the conditioned mixtures in separate containers designated for each test, thereby avoiding repeated conditioning of the same sample. Figure 5 and Figure 6 show the plant production process.

### 2.4. Testing Methods

The mechanical and performance properties evaluated in the plant-produced mixtures included cracking resistance, stiffness modulus, fatigue resistance, moisture susceptibility, and resistance to permanent deformation

**Figure 7:**
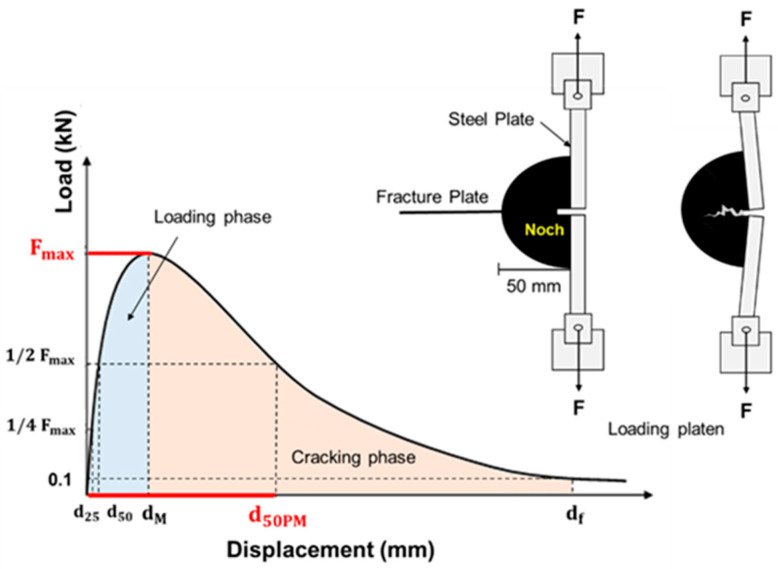
Fénix test setup and load–displacement output test curve.

Low-temperature cracking resistance was evaluated using the Fénix^®^ test, according to standard NLT 383/20 [55]. The test consisted of subjecting half of a cylindrical specimen to tension at a constant rate of 1 mm/min until fracture, as shown in Figure 7. Two steel plates were fixed to the flat face of the section, separated by an induced 5 mm crack across the diametral plane. The plates were attached to the testing press via a spherical joint, allowing free rotation of the load application points during the test. Force and displacement values were recorded simultaneously until the load dropped to 0.1 kN, which was the established criterion for test completion. Four specimens of each mixture type were tested at 0 °C and 10 °C.

The parameters evaluated were: maximum tensile force ($F_{max}$), maximum strength ($R_{T}$), tensile stiffness index (TSI), displacement at 50% of post-maximum tensile force ($d_{50PM}$), displacement between $d_{50PM}$ and $d_{M}$ ($D_{T}$), dissipated energy per unit area ($G_{D}$), and toughness index ($T_{I}$). The values of $R_{T}$, TSI, $G_{D}$ and $T_{I}$ were obtained from Equations (1), (2), (3), and (4), respectively.

(1) $$R_{T}=1000\times \frac{F_{max}\;}{S\;}$$

where $R_{T}$ is the Maximum strength in (Mpa). Fmax is the maximum load in (kN), and *S* is the fracture area in (m^2^).

(2) $$TSI=\frac{1/2F_{max}-1/4F_{max}}{(d_{50}-d_{25})\;}$$

where *TSI* is the Tensile stiffness index in (kN/mm). $F_{max}$ is the maximum load in (kN); $d_{50}$ is the displacement before maximum load at ½ $F_{max}$ in (mm); and $d_{25}$ is the displacement before maximum load at ¼ $F_{max}$ in (mm).

(3) $$G_{D}=\frac{\int_{0}^{df}F(x)\cdot dx}{S}$$

where $G_{D}$ is the Dissipated energy per unit area in (J/m^2^). F is the load in (kN); x is the displacement in (mm); df is the fracture displacement in (mm); and S is the fracture area in (m^2^).

(4) $$T_{I}=\frac{\int_{dM}^{df}F(x)\cdot dx}{S}\times (d_{50MP}-d_{M})$$

where $T_{I}$ is the toughness index is in (J/m^2^)·mm. $d_{M}$ is the displacement at Fmax in (mm), $d_{50PM}$ is the displacement after maximum load at ½ $F_{max}$ in (mm); and *S* is the fracture area in (m^2^).

The stiffness modulus ($S_{M}$) was determined by the indirect tensile strength test oflowing EN 12697-26, Annex C [56] and Equation (5). Cylindrical specimens were subjected to controlled horizontal deformation under a pulsating sinusoidal load, with an intermittent rest period. Four specimens per mixture type were fabricated and tested at temperatures of 5 °C, 20 °C, and 40 °C.

(5) $$S_{M}=\frac{F\times (v+0.27)}{\left(z\times h\right)}$$

where *S_M_* is the stiffness modulus measured in (MPa), *F* is the maximum vertical load applied in (N), v is Poisson’s ratio, z is the horizontal displacement in (mm), and h is the average thickness of the specimen in (mm).

**Figure 8:**
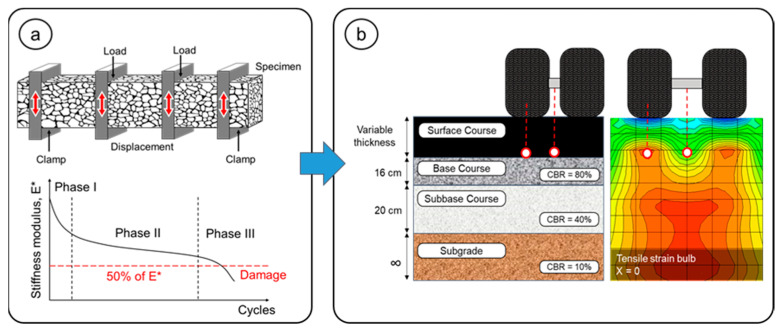
Resistance to fatigue damage: (**a**) 4PB Fatigue test; (**b**) empirical–mechanistic analysis.

Fatigue resistance of the asphalt mixtures was evaluated using the four-point bending (4PB) test following EN 12697-24, Annex D [57]. Prismatic beams (b = 50 mm; *h* = 50 mm; L = 400 mm) were subjected to repeated deformation-controlled loading until failure (Figure 8a), under a constant temperature of 20 °C. The tests were performed at a fixed frequency of 10 Hz, using three strain levels and a minimum of six replicates per level. HMA mixtures were tested at 170, 190, and 300 µε, whereas SMA mixtures were tested at 400, 500, and 700 µε. Fatigue curves were derived from the results through least squares regression using Equation (6):

(6) $$\varepsilon =a\;\times \;N^{-b}$$

where ε is the tensile strain, *N* is the number of cycles to failure, and a and b are coefficients of the fatigue laws.

An empirical–mechanistic analysis using PITRA PAVE was also performed to estimate deformations at critical pavement points (Figure 8b). Input parameters included the stiffness modulus at 20 °C, layer thickness, and a Poisson’s ratio of 0.35 for both mixture types. Two loading scenarios were considered, applied to a dual-wheel axle: 8.16 tonnes representing the standard design load, and 11 tonnes corresponding to the maximum allowable load in Chile for this axle type.

The sensitivity to moisture was evaluated by the indirect tensile strength ratio (ITSR), following the Chilean standard [50], adapted from ASTM D4867 [58]. ITSR values were determined using Equations (7) and (8). One subset was conditioned dry at 20 ± 5 °C, while the other was initially immersed in water under vacuum at 70 kPa, followed by conditioning in a water bath at 60 ± 1 °C for 24 h. Both subsets were subsequently conditioned at the test temperature of 25 °C in a water bath for one hour, ensuring that the dry subset did not contact water. Finally, all specimens were subjected to indirect tensile testing according to EN 12697-23 [59].

(7) $$ITS=\frac{\left(2\times P\right)}{\left(\pi \times D\times H\right)}\times 100$$

(8) $$ITSR=\frac{{ITS}_{w}}{{ITS}_{d}}\times 100$$

where ITS is the indirect tensile strength (kPa), P is the maximum applied load (kN), D is the specimen diameter (mm), H in the specimen height (mm), ${ITS}_{w}$ is the mean resistance to indirect tensile strength of the wet-conditioned test samples (Pa) and ${ITS}_{d}$ is the mean resistance to indirect tensile strength of the dry-conditioned test samples (Pa).

The resistance of the mixtures to permanent deformation was evaluated using the Hamburg wheel tracking test (HWTT) per AASHTO T 324 [60]. The test applied a repetitive load of 705 ± 4.5 N to cylindrical specimens submerged in a water bath at a controlled temperature of 50 ± 0.5 °C, over 10,000 cycles (equivalent to 20,000 passes). For each mixture type, four cylindrical specimens with a diameter of 150 mm were created using the Superpave gyratory compactor to ensure consistent density and compaction conditions.

The parameters evaluated included the average rut depth (RD), the percentage rut depth (PRD), and the slope of the deformation curve between cycles 5000 and 10,000 (WTS). The test also enabled assessment of stripping, manifested as a loss of adhesion between the asphalt binder and the aggregates, indicated by an increase in the slope of the deformation curve.

## 3. Results and Discussion

### 3.1. Design Properties of the Mixes

The design properties of the SMA mixtures are presented in Table 5. Regarding the SMA mixture design, it can be observed that both the reference mixture (SMA-R) and the mixture incorporating WTTF-based additive (SMA-F) exhibit very similar values for the evaluated design parameters. Both mixtures comply with the specifications established by the Chilean standard for SMA mixtures. In terms of binder drainage [58], both mixtures recorded values below 0.3%, meeting the requirements for this type of mixture. This indicates that the WTTF-based additive stabilizes the binder within the mixture matrix, preventing its drainage through the aggregate skeleton, confirming the laboratory-scale results reported by Valdés et al. (2022) [45].

The conditioning procedure applied prior to testing, along with the aging inherent to industrial production, likely contributed to asphalt binder oxidation, increasing mixture stiffness [61,62] and slightly affecting volumetric response during Marshall compaction, resulting in marginally higher air void contents. Nevertheless, SMA-R and SMA-F mixtures showed very similar air void contents, with all other parameters remaining consistent, indicating that these effects result from asphalt binder stiffening rather than the incorporation of the WTTF-based additive.

The design properties of the reference HMA mixture (HMA-R) are presented in Table 5. The results obtained, both in the laboratory and under industrial production, indicate consistent performance in accordance with Chilean specifications for HMA wearing courses. The industrially produced mixture shows an increase in Marshall stability and a reduction in flow compared with the laboratory-designed mixture. These results indicate an increase in the mixture’s structural stiffness, accompanied by a decrease in flexibility. As with the SMA mixtures, this behavior is attributed to asphalt binder aging associated with the mixture conditioning process.

### 3.2. Cracking Resistance

**Figure 9:**
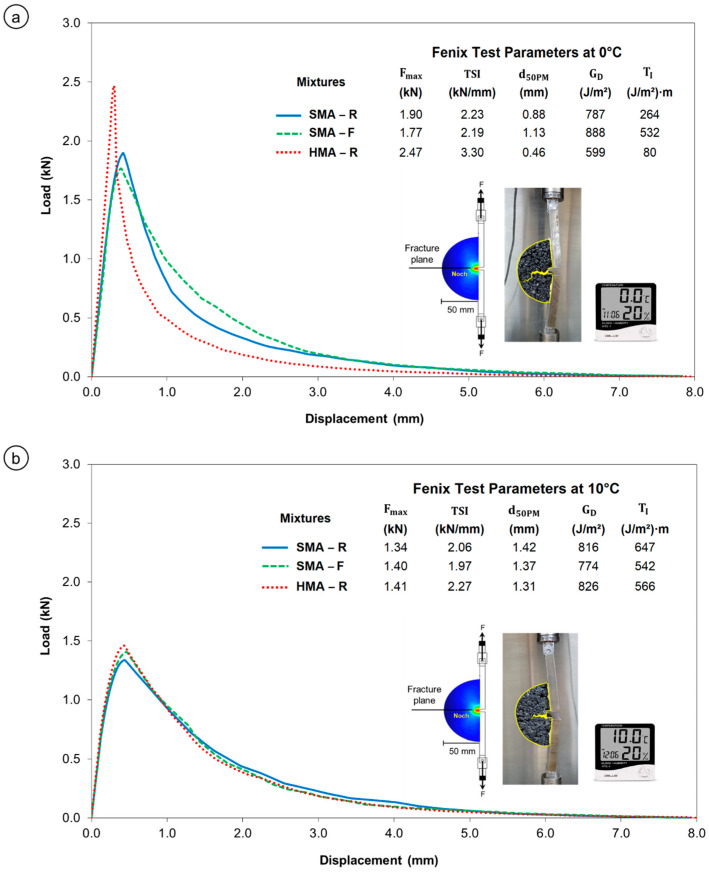
Fénix Test Stress–displacement Curves: (**a**) 0 °C; (**b**) 10 °C.

The cracking resistance of the asphalt mixtures was evaluated using parameters obtained from the Fénix test, which characterizes low-temperature cracking behavior. Figure 9a shows the load–displacement curves of the mixtures tested at 0 °C. At this temperature, the SMA-R and SMA-F mixtures exhibited similar maximum tensile force, with $F_{max}$, values of 1.90 kN and 1.77 kN, respectively. However, the SMA-F mixture showed a higher flexural capacity $d_{50PM}$, exceeding that of SMA-R and HMA-R by 22% and 59%, respectively.

This indicates greater ductility, defined as an increased deformation capacity prior to fracture, a key property in asphalt mixtures subjected to low temperatures, as it helps delay crack formation [63]. This trend is consistent with laboratory observations [45], where SMA-R and SMA-F maintained similar $F_{max}$ values, while $d_{50PM}$ was 9% higher for the SMA-F mixture.

**Figure 10:**
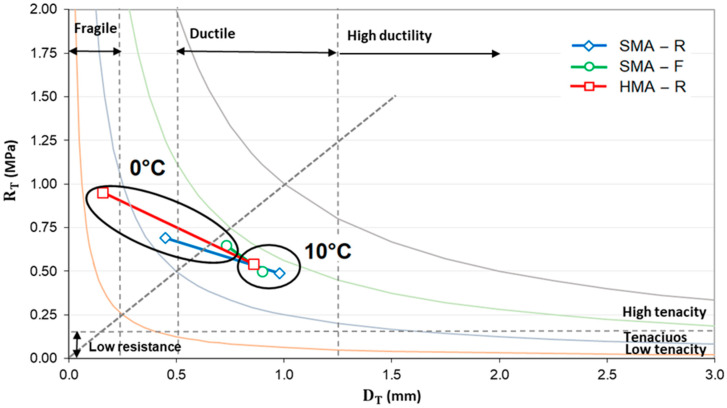
Fénix Stress–Strain Diagram©. Temperatures 0 °C and 10 °C. (The Fénix diagram is associated with a test methodology. 2019 UPC All rights reserved).

Furthermore, the SMA-F mixture exhibited higher dissipated energy and toughness index ($T_{I}$) than SMA-R, indicating greater toughness and an improved capacity to dissipate energy in the post-peak softening region. These results are consistent with laboratory observations [45], where SMA-F recorded $G_{D}$ and $T_{I}$ values 11% and 19% higher than SMA-R, respectively. Figure 10 presents the Fénix© stress–strain diagram, showing that SMA-F tested at 0 °C displayed increased ductility while achieving maximum tensile resistance values comparable to SMA-R, confirming enhanced deformation capacity without compromising structural strength. These effects may be related to the reinforcement provided by the end-of-life tire fiber (WTTF) within the asphalt mixture matrix, which absorbs and dissipates stresses [22,39,41].

On the other hand, the HMA-R mixture exhibited more brittle behavior, with a lower capacity for deformation and energy dissipation, increasing its susceptibility to low-temperature cracking. In contrast, the SMA mixtures demonstrated superior performance due to their characteristic design, featuring a high concentration of coarse aggregates, higher asphalt content, and fibers that contribute to toughness and cohesion [21,64,65].

Figure 9b shows the stress-displacement curves of the mixtures evaluated at 10 °C. At this temperature, the SMA-F mixture exhibited behavior similar to that of SMA-R and HMA-R with respect to the mechanical parameters associated with asphalt mixture cracking, as confirmed by the Fénix© Stress–Strain Diagram (Figure 10). This behavior is consistent with laboratory observations, where at 10 °C the mechanical parameters of SMA-R and SMA-F were comparable [45]. Furthermore, all mixtures tested at 10 °C displayed more ductile behavior than at 0 °C, indicating a greater capacity for deformation prior to fracture and, consequently, lower susceptibility to thermal cracking.

### 3.3. Stiffness Modulus

**Figure 11:**
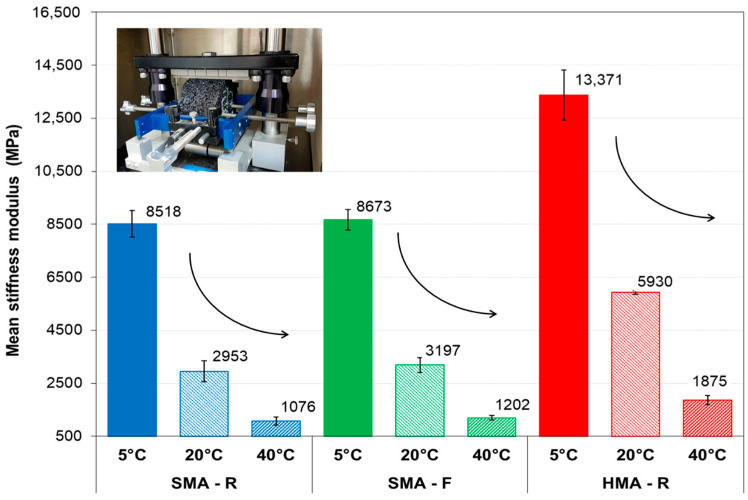
Stiffness modulus of the SMA mixtures studied at 5 °C, 20 °C, and 40 °C.

Figure 11 presents the stiffness modulus values obtained for the mixtures evaluated at 5 °C, 20 °C, and 40 °C. The results indicate a progressive decrease in stiffness modulus with increasing temperature, consistent with the viscoelastic behavior of the asphalt binder.

At 5 °C, the SMA-R and SMA-F mixtures exhibited similar stiffness modulus values. In contrast, the HMA-R mixture showed a notable increase of approximately 57% and 54% compared with SMA-R and SMA-F, respectively. These findings are consistent with the Fénix test results, which indicated higher stiffness for HMA-R at low temperatures and greater ductility in the SMA mixtures.

At 20 °C, the same trend was observed, with very similar stiffness modulus values for SMA-R and SMA-F, both close to 3000 MPa. This is consistent with laboratory observations [45], where the use of the WTTF-based additive did not affect the mechanical response of SMA-R in terms of stiffness. This behavior aligns with several studies indicating that mineral and synthetic fibers contribute to the cohesion of the aggregate-binder matrix [22,41,66], which may explain why the mixture is not negatively affected in this property. In contrast, HMA-R exhibited approximately twice the stiffness of SMA-R and SMA-F. This is noteworthy, considering that the most frequent thermal conditions during pavement service life are around 20 °C, under which repeated vehicular loading increases the likelihood of fatigue cracking [67].

At 40 °C, all evaluated mixtures exhibited a significant reduction in stiffness values, while maintaining the same trend observed at lower temperatures. The HMA-R mixture yielded the highest stiffness, followed by SMA-F and SMA-R. This reduction is explained by the loss of binder consistency at elevated temperatures, leading to greater deformation [13].

The observed stiffness differences between SMA and HMA mixtures are attributed to their design characteristics. SMA mixtures, due to their higher binder content, exhibit greater ductility, whereas HMAs, with lower binder content and a more compact structure, display higher stiffness and more brittle mechanical behavior.

### 3.4. Fatigue Resistance

**Figure 12:**
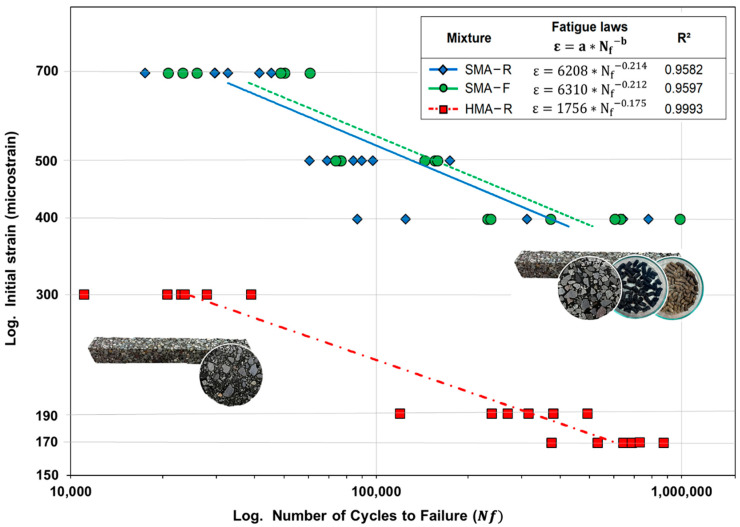
Fatigue laws of the evaluated mixtures obtained by the four-point bending test.

The fatigue laws obtained from the four-point bending (4PB) test are shown in Figure 12. The results indicate that the SMA-F mixture exhibited a higher number of load cycles than the SMA-R mixture for the same strain levels, demonstrating an increased capacity to resist accumulated damage under repeated loading. At strain levels of 700 µε, 500 µε, and 400 µε, the SMA-F mixture showed an increase in fatigue life of 17%, 19%, and 20%, respectively, compared to the SMA-R mixture.

This improvement is likely associated with the inclusion of polymeric fibers, which, as reported by Guo et al. (2020) [68], are capable of slowing crack development and prolonging the elastic response phase in asphalt mixtures. These results align with the findings of Mahrez and Karim (2010) [69], who reported increases in fatigue life of between 10% and 79% compared with the control mixture when glass fibers were incorporated into the SMA mixtures.

The fatigue performance observed in the plant-produced SMA-F mixture is consistent with laboratory findings [46], where increases of up to 63% in fatigue cycles were reported at equivalent strain levels relative to SMA-R. The smaller improvement measured in the plant-produced mixtures (17–20%) can be attributed to the additional thermo-oxidative aging induced during industrial production, in addition to the conditioning process required to restore the workability necessary to shape the beams for the 4PB test. This aging process typically increases binder stiffness and reduces its capacity to dissipate energy, accelerating fatigue damage [70]. Despite this additional aging, the SMA-F mixture maintained superior mechanical performance compared to SMA-R, suggesting that the WTTF may mitigate aging-induced embrittlement. Similar behavior has been reported by Cao et al. (2025) [71] in other fiber-reinforced asphalt systems, where bamboo fibers preserved viscoelastic behavior and fatigue resistance more effectively than lignin fibers under laboratory aging. A comparable effect may be occurring in the present study: unlike the cellulose stabilizer used in SMA-R, the WTTF could help maintain a more favorable balance between stiffness and flexibility after aging, which contributes to the improved fatigue endurance observed in SMA-F despite the harsher aging conditions associated with plant production.

In the case of the HMA-R mixture, lower strain levels (170 µε, 190 µε, and 300 µε) were applied compared with the SMA-R and SMA-F mixtures, due to their lower ductility. The results show that, at strain levels 50–60% lower than those applied to the SMA mixtures, HMA-R exhibited a similar number of cycles to failure. This indicates that the SMA mixtures were less susceptible to fatigue cracking, requiring higher stress levels to reach the same failure condition as HMA-R. Determination coefficients (R^2^) between 0.95 and 0.99 demonstrated a strong correlation between initial strain and fatigue performance in the asphalt mixtures tested.

The results of the fatigue parameters, including initial phase angle, final phase angle, and dissipated energy, obtained for the mixtures tested in the 4PB test, are presented in Table 6. The phase angle (δ) increased with strain, reaching an average close to 45° in the SMA-R and SMA-F mixtures, as previously observed in laboratory studies [46]. This behavior indicates a balanced viscoelastic response of the asphalt binder, which promotes energy dissipation during load cycles and delays the propagation of microcracks associated with fatigue cracking [72]. Conversely, the HMA-R mixture exhibited a more pronounced elastic response, with reduced energy dissipation, leading to higher stress concentrations and accelerated fatigue crack development.

The evaluation of dissipated energy indicates that the SMA-F mixture exhibited the highest accumulated energy before failure, followed by SMA-R and HMA-R. This greater capacity to absorb and dissipate energy reflects the superior resistance of the SMA-F mixture to fatigue damage, as it requires more mechanical work and a higher number of load cycles to reach failure, explaining the observed increase in cycles to failure. These results are consistent with laboratory observations [46] and align with Benavides et al. (2024) [73], who reported that higher accumulated dissipated energy is associated with an increased ability of the material to accommodate deformations, resulting in improved fatigue resistance. The enhanced fatigue and cracking resistance observed in the SMA-F mixture may be associated with interfacial mechanisms related to the morphology of the waste tire textile fibers (WTTF). These fibers retain adhered rubber granules and fine crumb particles from the ELT processing stage (Figure 2), which increase their surface roughness and specific surface area. This roughened morphology promotes stronger mechanical interlocking and improved interfacial cohesion with the asphalt binder, facilitating more efficient stress transfer under repeated loading. Similar behavior was reported by Xie et al. (2024) [74], who observed that increasing the surface roughness of PET fibers through graphene pretreatment significantly enhanced the fatigue resistance of modified asphalt binders. In the present study, the adhered rubber particles on WTTF likely produced an analogous reinforcing effect, contributing to the improved resistance to cracking and fatigue observed in the fiber-stabilized SMA mixture. This mechanism is also consistent with evidence indicating that the interfacial transition zones (ITZs) between fiber-asphalt and binder-aggregate are critical weak regions where failure commonly initiates and propagates. In this context, Jiu et al. (2025) [75] report that FTIR analyses show fiber-binder and aggregate-binder interactions are predominantly governed by physical adsorption rather than significant chemical bonding, further underscoring the importance of surface morphology in governing interfacial performance.

**Figure 13:**
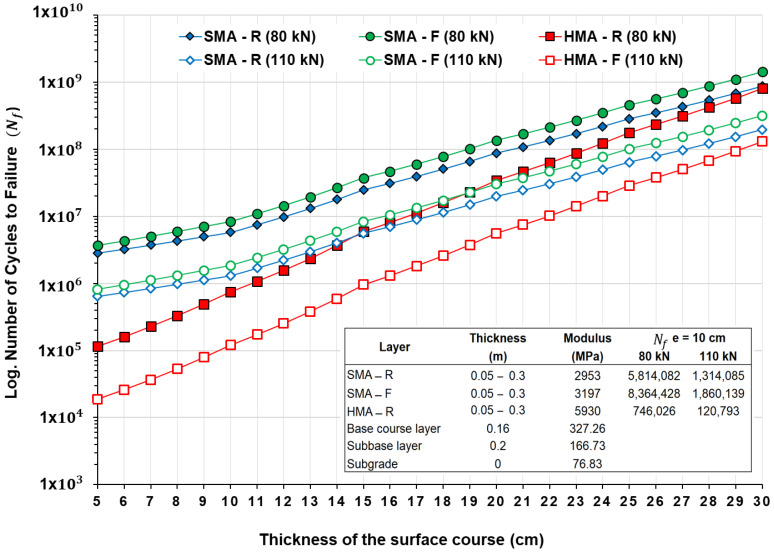
Pavement structure durability of evaluated mixtures for different thicknesses and traffic loads.

The pavement structural durability for the evaluated mixtures, considering different asphalt layer thicknesses and two-axle loads of 80 kN and 110 kN, is presented in Figure 13. For a given layer thickness, the SMA mixtures were able to withstand a higher number of cycles before failure, indicating an extended fatigue life. This behavior is primarily attributed to the inherent characteristics of SMA mixtures [76]. For an average thickness of 10 cm, the SMA-F mixture under an 80 kN load exhibited an increase in durability of approximately 44% compared with the SMA-R mixture. This trend persists under higher loads of 110 kN, with an increase of around 42%. These results demonstrate the effectiveness of the WTTF-based additive in enhancing pavement durability, consistent with laboratory findings [46], where SMA-F durability was observed to increase up to twice that of SMA-R. The larger magnitude of this increase under laboratory conditions is explained by the additional aging experienced by plant-produced mixtures during conditioning.

Additionally, when evaluating durability specifically for 10,000,000 load cycles, it is observed that the use of the end-of-life tire fiber (WTTF) in the SMA-F mixture would allow a reduction of 1.0 to 1.5 cm in the wearing course thickness without compromising the fatigue performance of the SMA-R mixture, consistent with laboratory results [46]. Regarding the HMA-R mixture, lower durability was observed at both load levels, in agreement with the fatigue law results, which showed the least favorable condition in terms of fatigue resistance. This behavior is associated with the stiffness modulus characteristics and the response observed in the fatigue laws. The increased stiffness modulus at 20 °C in the HMA-R mixture indicates higher structural rigidity, resulting in greater susceptibility to cracking, as reflected in the fatigue law outcomes.

### 3.5. Resistance to Moisture Damage

**Figure 14:**
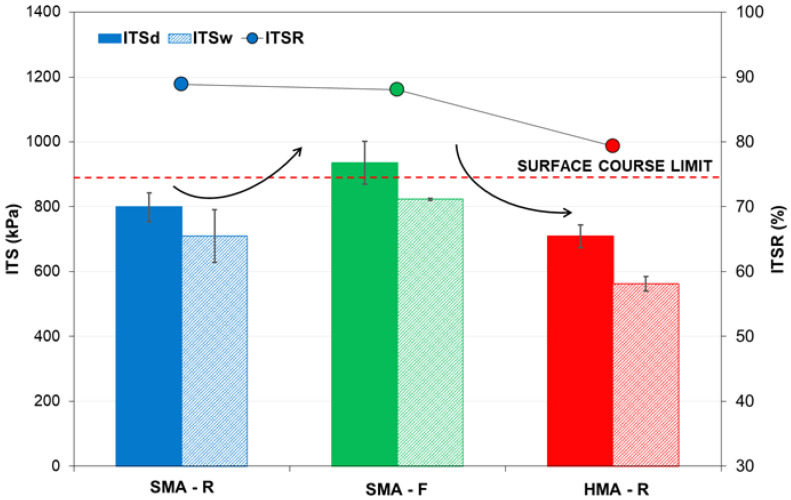
Moisture sensitivity test results.

The indirect tensile strength of the evaluated mixtures under dry (${ITS}_{d}$) and wet (${ITS}_{w}$) conditions is shown in Figure 14. The results indicate the superior performance of the SMA-F mixture, with approximate increases of 18% in ${ITS}_{d}$ and 17% in ${ITS}_{w}$ compared to the SMA-R mixture. This behavior reflects a lower moisture susceptibility of the SMA-F mixture, resulting in greater resistance to water action. These findings are consistent with laboratory observations [45], where the WTTF-based additive demonstrated good resistance to moisture-induced damage, maintaining performance comparable to SMA-R. This improvement in performance can be attributed to the incorporation of waste tire polymer fibers in the SMA-F mixture, in agreement with the study by Yin and Wu (2018) [24], who observed enhanced moisture resistance in SMA mixtures containing synthetic nylon fibers. Furthermore, various studies have evaluated other types of polymer fibers in asphalt mixtures, reporting significant improvements in resistance to moisture-induced damage [77,78,79].

Regarding the HMA-R mixture, the results indicated lower indirect tensile strength under both dry $\left({ITS}_{d}\right)$ and wet $({ITS}_{w}$) conditions, compared with SMA-F and SMA-R. This behavior demonstrates that HMA-R exhibits higher moisture susceptibility, increasing its likelihood of moisture-related damage relative to the SMA mixtures. This greater vulnerability can be attributed to the lower asphalt binder content in HMA-R, which reduces aggregate adhesion and facilitates water infiltration, compromising both structural integrity and mixture durability. Despite these differences in performance, all mixtures met the minimum indirect tensile strength ratio (ITSR) required by Chilean specifications for surface courses (ITSR > 75%). The SMA-R and SMA-F mixtures achieved higher ITSR values, reflecting a lower likelihood of adhesive failure (stripping) and improved resistance to moisture damage.

### 3.6. Resistance to Permanent Deformation

**Figure 15:**
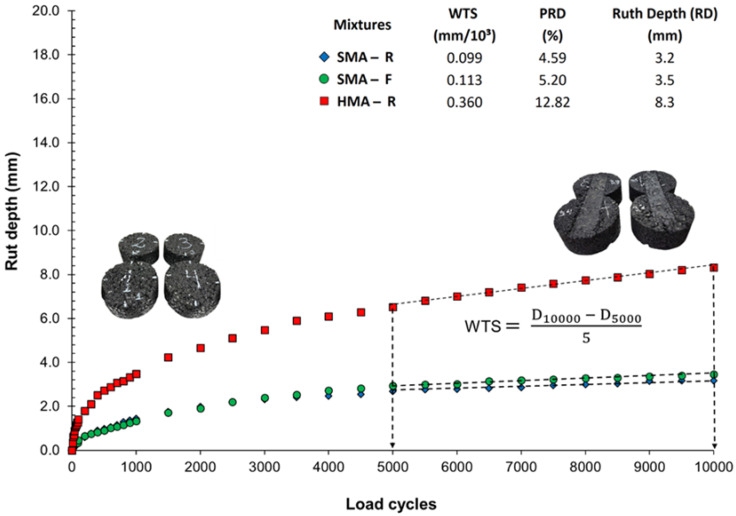
Hamburg wheel tracking test results at 50 °C.

The evolution of rut depth (RD) with the number of load cycles, obtained from the Hamburg Wheel Tracking Test, is presented in Figure 15 results showed that both SMA-R and SMA-F exhibited a low deformation rate, with differences in RD of only 0.3 mm at 10,000 cycles. This indicates that the addition of polymer fiber-based additive in SMA-F had no significant effect on permanent deformation resistance, maintaining a mechanical performance similar to that of SMA-R. These findings are consistent with laboratory observations [45], where the incorporation of various percentages of waste tire polymer fibers (WTTF) in SMA resulted in RD values close to 3.4 mm, with a maximum difference of 0.6 mm.

In contrast, the HMA-R mixture showed significantly greater RD, exceeding those of SMA-R and SMA-F by 159% and 137%, respectively. This highlights its reduced ability to resist permanent deformation under repeated high-temperature loading. The observed behavior can be attributed to fundamental structural differences between HMA and SMA mixtures, with SMA distinguished by a dense aggregate skeleton and the use of polymer-modified binder, both of which enhance matrix cohesion and contribute to improved rutting resistance [21,64,65].

Additionally, the deformation slopes (WTS) between cycles 5000 and 10,000 revealed that SMA-R and SMA-F exhibited lower slopes compared with HMA-R, indicating a slower progression of damage. None of the mixtures showed evidence of stripping, typically manifested as an abrupt change in the slope of the curve. These findings are consistent with the ITSR results, confirming good resistance of the mixtures to moisture-induced damage.

## 4. Conclusions

The additive based on polymer fibers derived from end-of-life tires (WTTF) can be employed as a stabilizing agent in the industrial production of SMA mixtures, ensuring compliance with design requirements and enhancing the mechanical performance of the mixture.

The results obtained for the industrially produced SMA-F mixture are consistent with those from laboratory tests, showing superior mechanical performance in terms of low-temperature cracking resistance, fatigue resistance, and moisture damage resistance. At the same time, stiffness modulus and permanent deformation behavior were similar to those of SMA-R. This confirms that the laboratory results are scalable to the industrial production of SMA mixtures incorporating the WTTF-based polymer additive.

Evaluation of the volumetric and mechanical design properties indicated that replacing the commercial cellulose additive in SMA-R mixtures with the WTTF-based additive effectively maintained the design specifications, satisfying the parameters established for wearing course layers. Moreover, the WTTF-based additive contributed to stabilizing the SMA mixture, resulting in binder drain-down values below the prescribed limits. Similarly, the HMA-R mixture complied with all requirements for use in wearing course applications

Regarding low-temperature cracking resistance at 0 °C, SMA-F exhibited higher flexural capacity ($d_{50PM}$), dissipated energy ($G_{D}$), and toughness ($T_{I}$), while maintaining a similar maximum tensile strength $(F_{max}$) to SMA-R. This behavior indicates an improved capacity to absorb deformations without compromising structural integrity, providing better resistance to thermal cracking. Additionally, at low temperatures, SMA-F demonstrated greater ductility and lower thermal susceptibility. In contrast, HMA-R exhibited more brittle behavior with lower deformation and energy dissipation, resulting in higher vulnerability to thermal cracking. At 10 °C, all asphalt mixtures displayed comparable mechanical performance.

With respect to the stiffness at 5 °C, 20 °C, and 40 °C, the WTTF-based polymer additive did not affect the stiffness modulus of SMA-R, showing similar behavior. HMA-R, however, exhibited higher stiffness values across the evaluated temperatures, indicating greater rigidity.

Fatigue laws and durability analyses of the conditioned mixtures revealed that SMA-F demonstrated superior resistance to fatigue damage compared with SMA-R and HMA-R, showing a higher capacity to withstand load cycles before failure and an increase in durability of up to 44% relative to SMA-R.

Moisture sensitivity tests showed that SMA-F achieved higher indirect tensile strength (ITS) under both dry $\left({ITS}_{d}\right)$ and wet $\left({ITS}_{w}\right)$ conditions than with SMA-R. HMA-R displayed greater susceptibility to moisture-induced damage. All evaluated mixtures met the ITSR limits required by Chilean standards for wearing course layers (>75%).

Permanent deformation resistance exhibited similar behavior between the SMA-F and SMA-R mixtures in terms of rutting depth (RD). Both mixtures displayed a low deformation rate compared with HMA-R, which showed greater rutting depth and lower resistance to permanent deformation. No mixture exhibited stripping.

The results obtained confirm the feasibility and scalability of using the WTTF-based polymer additive in industrial processes. This validation provides a foundation for progressing towards the construction of a trial section and for monitoring its in-service performance under different traffic demands and climate conditions, representing a crucial step for the practical implementation and optimization of improved asphalt pavement design.

## Figures and Tables

**Table 1:** Properties of the asphalt binder used in SMA mixtures.

Tests	CA 60/80	Specs. (*)	Methods
Penetration at 25 °C, 100 g. 5 s. (0.1 mm)	66	60–80	8.202.3 (*)
Softening point R&B (°C)	71.4	Min. 65	8.302.16 (*)
Ductility at 25 °C, 5cm/min (cm)	100	Min. 80	8.302.8 (*)
Linear elastic recovery at 13 °C, 20 cm, 1 h (%)	83	Min. 60	8.302.19 (*)
Elastic recovery by torsion at 25 °C (%)	82	Min. 70	NLT 329 [51]
Penetration index	3.8	Min. +3.0	8.302.21 (*)
FRAASS breaking point (°C)	<−15	Max. −15	8.302.17 (*)
Flash point (°C)	>235	Min. 235	8.302.9 (*)
Storage stability	0.7	Max. 5	ASTM D5892 [52]
Density pycnometer at 25 °C (kg/m^3^)	1010	To be reported	8.302.2 (*)
RTFOT (Rolling Thin-Film Oven Test)			
Mass loss (%)	0.4	Max. 1.0	8.302.33 (*)
Penetration (% original) (%)	77	Min. 60	8.302.3 (*)
Softening point variation (°C)	0.6	−5.0 to +10	8.302.16 (*)
Mixing temperature at 2 Poise (°C)	181 ± 5	To be reported	
Compaction temperature at 3 Poise (°C)	169 ± 5	To be reported	

(*) Chilean norms [50].

**Table 2:** Properties of the asphalt binder used in HMA mixtures.

Tests	CA-24	Specs. (*)	Methods (*)
Absolute viscosity at 60 °C, 300 mm Hg (P)	2578	Min. 2400	8.202.15
Penetration at 25 °C, 100 g. 5 s. (0.1 mm)	60	Min. 40	8.202.3
Ductility at 25 °C, 5 cm/min (cm)	>100	Min. 100	8.202.8
Spot test (hep-xyl) 30% xylene	Negative	Negative	8.302.7
Cleveland open cup flash point (°C)	>232	Min. 232	8.302.9
Softening point R&B (°C)	51	To be reported	8.202.16
Trichloroethylene solubility (%)	>99	Min. 99	8.302.11
Penetration index	−0.5	−2.0 to +1.0	8.202.18
Density pycnometer at 25 °C (kg/m^3^)	1015	To be reported	8.302.2
RTFOT (Rolling Thin-Film Oven Test)			
Mass loss (%)	0.1	Max. 0.8	8.302.33
Absolute viscosity at 60 °C, 300 mm Hg (P)	7950	To be reported	8.302.15
Ductility at 25 °C, 5 cm/min (cm)	>100	Min. 100	8.202.8
Durability index	3.1	Max. 4.0	8.301.1
Mixing temperature at 2 Poise (°C)	154 ± 5	To be reported	
Compaction temperature at 3 Poise (°C)	142 ± 5	To be reported	

(*) Chilean norms [50].

**Table 3:** Physical properties of aggregates used.

Tests	SMA	HMA	Specs. (*)	Methods (**)
Coarse aggregate				
Crushed aggregates (%)	100	98	Min. 90	8.202.6
Flaky particles (%)	0.5	0.3	Max. 10	8.202.6
Flakiness index (%)	12	13	Max. 25	8.202.7
Los Angeles abrasion loss (%)	14	14	Max. 25	8.202.11
Sodium sulfate soundness (%)	0.3	0.3	Max. 12	8.202.17
Boil method adhesion	≥95	N/A	Min. 95	8.302.59
Static method adhesion	≥95	≥95	Min. 95	8.302.29
Dynamic method adhesion	≥95	≥95	Min. 95	8.302.31
Fine aggregate				
Plasticity index	Non-plastic	Non-plastic	Non-plastic	8.102.4
Riedel-Weber adhesion	5–10	3–8	Min. 0–5	8.302.30
Soundness sodium sulfate (%)	1.0	1.0	Max. 15	8.202.17
Combined aggregate				
Soluble salts (%)	0	0	Max. 2	8.202.14
Sand equivalent (%)	52	54	Min. 50	8.202.9
Water absorption (%)	1.22	N/A	Max. 2	8.202.20 8.202.21

(*) Chilean norms [53]. (**) Chilean norms [50].

**Table 4:** Virgin aggregates gradation used in asphalt mixtures.

Sieve Size		SMA Mixtures				HMA Mixtures			
		Gradation (% Passing)				Gradation (% Passing)			
mm	ASTM	1/2”	3/8”	1/4”	Filler	3/4”	1/2”	1/4”	Filler
19.0	3/4”	100	100	100	100	100	100	100	100
12.5	1/2”	100	100	100	100	40	100	100	100
11.2	7/16” (**)	89	100	100	100	–	–	–	–
9.5	3/8”	67	100	100	100	3	83	100	100
8.0	5/16” (**)	45	86	100	100	–	–	–	–
6.3	1/4” (**)	18	40	100	100	–	–	–	–
5.6	N°3.5 (**)	9	10	100	100	–	–	–	–
4.75	N°4	3	6	95	100	1	4	94	100
2.36	N°8	1	3	68	100	1	1	64	100
2.0	N°10 (**)	1	1	60	100	–	–	–	–
1.18	N°16 (*)	–	–	–	–	1	1	46	100
0.6	N°30	1	1	32	100	1	1	34	100
0.3	N°50	1	1	24	100	1	1	25	99
0.15	N°100	1	1	17	96	1	1	19	93
0.075	N°200	1	1	14	81	1	1	14	77
0.063	N°230 (**)	1	1	13	76	–	–	–	–

(*) Sieve not included in the SMA mixture design. (**) Sieve not included in the HMA mixture design.

**Table 5:** Design parameters of the SMA and HMA mixtures.

STONE MASTIC ASPHALT (SMA)										
Mix Type	Manufacturing Temperature	OAC (*)	WBA (**)	CCA (***)	Density	Air Voids	VMA	${VCA}_{MIX}$	${VCA}_{DRC}$	Binder Drain Down
	(°C)	(wt% of Aggregate)	(wt% of Aggregate)		(kg/m^3^)	(%)	(%)	(%)	(%)	(%)
**Asphalt mixtures design**										
SMA-R	176	7.3	0	0.5	2319	4.5	19.4	30.3	41.6	0.12
SMA-F	176	7.3	0.5	0	2316	4.5	19.5	30.3	41.6	0.18
**Verification of industrial asphalt mix designs**										
SMA-R	176	7.3	0	0.5	2300	5.7	19.6	30.2	51.6	–
SMA-F	176	7.3	0.5	0	2279	6.6	20.2	30.2	41.6	–
Chilean Specifications for surface course [54]						4	Min. 17	${VCA}_{MIX}$ < ${VCA}_{DRC}$		Max. 0.3
**HOT MIX ASPHALT**										
Mix type	Manufacturing Temperature (°C)	OAC (*) (wt% of aggregate)			Density (%)	Air voids (%)	VMA (%)	Stability (N)	Flow 0.25 mm	
**Asphalt mixtures design**										
HMA-R	152	5.1			2388	4.4	14.9	13061	14.7	
**Verification of industrial asphalt mix designs**										
HMA-R	152	5.1			2406	4.0	14.3	13351	11.0	
Chilean specifications for surface course [53]						3–5	Min. 14	Min. 9000	8–16	

(*) OAC: Optimum asphalt content. (**) WBA: WTTF-based additive. (***) CCA: Cellulose commercial additive.

**Table 6:** Results of the parameters obtained in the four-point bending test.

Mix Type	Initial Strain	Fatigue Cycles	Initial Phase Angle	SD (*)	Final Phase Angle	SD (*)	Cumulative Dissipated Energy	SD (*)
	(µε)	$\left(N_{f}\right)$	(δ)		(δ)		(J/m^3^)	
SMA – R	700	32,707	44.0	1.3	52.2	1.5	36.7	9.2
	500	95,902	40.4	0.8	48.3	0.8	61.3	22.3
	400	426,202	39.2	0.7	46.7	0.8	174.0	109.2
SMA – F	700	38,259	43.0	1.0	50.9	1.0	44.6	18.9
	500	114,184	40.4	0.5	47.9	1.0	72.2	25.6
	400	510,442	39.7	1.1	44.1	5.7	207.1	127.2
HMA – R	300	24,180	32.1	2.9	42.3	0.8	12.1	4.2
	190	302,341	27.4	4.1	31.5	5.4	57.6	20.8
	170	639,508	26.0	0.8	29.8	3.4	91.5	25.6

(*) Standard Deviation.

## Data Availability

The original contributions presented in this study are included in the article. Further inquiries can be directed to the corresponding author.
